# E-inclusion: Beyond individual socio-demographic characteristics

**DOI:** 10.1371/journal.pone.0184545

**Published:** 2017-09-14

**Authors:** Patrícia Silva, Alice Delerue Matos, Roberto Martinez-Pecino

**Affiliations:** 1 Communication and Society Research Centre, Institute of Social Sciences, University of Minho, Braga, Portugal; 2 Department of Sociology and Communication and Society Research Centre, Institute of Social Sciences, University of Minho, Braga, Portugal; 3 Department of Social Pyschology, Universidad de Sevilla, Seville, Spain; Fondazione Ugo Bordoni, ITALY

## Abstract

The changing demographic structure of the population, resulting in unparalleled growth of the elderly population, means that e-inclusion of this population group is considered to be a social and political priority in the context of the Information Society. Most research studies have only considered individual variables -such as age, gender, education, income and health- in the explanatory models of e-inclusion of senior citizens, while ignoring macro variables, such as the welfare systems and public policies in each country. Simultaneously, most studies focus on small-scale samples, lack international comparisons and do not consider the combined effect of several variables that influence Internet use. This study aims to analyse possible differences between two countries that have different welfare systems and public policies, after controlling for the effects of the individual variables that have been identified in the literature as relevant for Internet use. The study focuses on a sample of 8639 individuals, aged 50 years and over, residing in Portugal and Estonia, who participated in the SHARE project (*Survey of Health*, *Ageing and Retirement in Europe*). The results of the logistic regression analysis demonstrate that welfare systems and public policies have an impact on the likelihood of Internet use, thus reinforcing the importance of developing public policies to foster e-inclusion of senior citizens.

## Introduction

The Internet has redesigned our daily lives, blurring the boundary between the local and the global, in terms of fostering access to knowledge and information, and disseminating ideas and values. It has also changed the way that people interact, and the manner in which they build their social networks [1–3]. However, some individuals remain on the margins of this "revolution" [4]. The "digital divide" between those who have access to information and communication technologies (ICT) and those who don’t, has concerned the public authorities, given that it is an obstacle to achieving the Information Society, which is considered to be economically more competitive and that fosters greater social cohesion, participation and control of citizens [5–7].

Studies on the "digital divide" identify senior citizens as one of the groups most vulnerable to e-Exclusion [3]. This "digital divide" results from inequalities in Internet access, types of use of Internet technology, knowledge of its technical features, ability to assess information quality and, among other factors, the diversity of forms of use [4]. Indeed, there is a major political, social and economic interest in identifying the factors that affect Internet use in order to avoid the "digital divide" and, in particular, to avoid the marginalization of senior citizens, given that they constitute a fast-growing population group [8].

Despite the relevance of the “digital divide”, scientific literature still lacks a comprehensive explanation of technology acceptance and, in particular, internet’ adoption by the elderly [9]. Studies of older age groups have shown, in general, that certain socio-demographic characteristics of individuals -such as age, gender, education, health status and income—condition the use of technology [10–19]. The influence of socio-demographic variables on Internet use has been shown by studies based on different technology acceptance models -like the Unified Theory of Acceptance and Use of Technology (UTAUT)- (e.g.[9,20]).

Literature evidences the importance of individual socio-demographic aspects employing qualitative as well as quantitative methodologies. In this sense studies using qualitative methodologies have focused in-depth on small sample sizes ranging from 10 to 24 participants [21–23] or slightly bigger [24]. Studies with quantitative methodologies, mainly surveys, also analyze the relevance of individual socio-demographic characteristics such as: age, gender, education [25–27], income [25–27], health [25,27] in the USA, Europe and worldwide (e.g. [25,26,28,29]). All in all, these studies confirm the influence of individual socio-demographic characteristics on information technology use. (For more detail see table in annex [4,19,25–41]).

On the other hand, several authors argue that inequalities in ICT use, when observed individually, are no more than a reflection of inequalities in social structures [42], and are therefore related to the economic, political, historical and social characteristics of the respective countries [43]. In this vein, there is a need for comparative studies between countries with different welfare regimes to evidence the extent of these factors [44]. Analyses with large sample sizes, that include a comparison between countries considering the simultaneous effect of various different factors, as in this work, are particularly important and needed since it is thereby possible to discuss the possible effect of different policies and welfare systems. Taking into consideration the literature review conducted, the main objective of this work was to analyse the existence of eventual differences in Internet use amongst individuals aged 50 years and over, resident in countries with different welfare systems and public policies, after having controlled for the socio-demographic variables that are identified in the literature as being important to explain Internet use.

## Materials and methods

This study focuses on 8639 individuals, aged 50 years and over, interviewed in wave 4 of the European project, SHARE (*Survey of Health*, *Ageing and Retirement in Europe*), in two countries with different welfare systems and public policies: Portugal and Estonia.

Details on the SHARE study in Europe have been described elsewhere[45]. Briefly, in wave 4 (2010–2011), a survey was conducted with representative samples of the non-institutionalised population aged 50 + in 16 European countries.

To achieve representation of this population, SHARE employs a sample design which involves baseline samples of the household population aged 50 and older at a particular point in time in each country, supplemented by regular refreshment samples of the sub -population of individuals who have turned 50 since the original baseline sample was selected [45].

Interviews were face-to-face and took place in the household. Trained interviewers conducted interviews using exactly the same questionnaire in all countries on a computer assisted personal interviewing program (CAPI).

The SHARE project, coordinated internationally by the Max Planck Institute for Social Law and Social Policy (Germany), has been approved by the Ethics Council of the Max-Planck-Society for the Advancement of Science and by the Ethics Committees of the institutions responsible for the study in the participating countries.

Given that the SHARE project has national samples of different sizes (in this study Portugal N = 1972 and Estonia N = 6667) and a sample design that is not uniform in the different countries, calibrated individual weights were used in the descriptive statistical analysis that aimed to present a comparative study of regular Internet users, and non-users. We used the chi-square test to assess the interdependence between the two qualitative variables. The sample means were also compared using Student’s t-tests for independent samples. Statistical test results with p < .05 were considered to be significant. The results from these tests were also complemented with effect size measures (Cohen’s d/Phi). The interpretation of results was based on Cohen (1988)[46].

In order to identify the determinants of Internet use, a binary logistic regression analysis, using the Enter method, was subsequently carried out. These analyses were performed using SPSS software, version 23.

The following variables and corresponding methods were used in this study:

Internet use: a dichotomous variable related to regular use of the Internet in order to send and receive e-mails, or for other purposes such as shopping, browsing information or making travel reservations. This variable has the status of a dependent variable in binary logistic regression analysis -wherein an affirmative answer to the question on Internet use is the reference category.

Socio-demographic variables: age; gender; number of years of schooling and self-perception of financial stress. In the latter case, this variable distinguished between individuals who reported that they experienced "major" or "some difficulties" in paying monthly expenses, and those who claimed that it is "easy" or "very easy" to meet such expenses, according to their incomes.

Health variables: mental health was assessed using the EURO-D scale and included a category that encompassed individuals who reported significant symptoms of depression, i.e. who obtained a score of equal to or higher than 3 points in the Euro-D 12-items scale [47] and another that encompasses individuals who recorded lower scores.

Physical health was addressed using two variables: the ADL scale and mobility limitations. The first variable, i.e. the ADL scale (limitations with activities of daily living), evaluates the functional dependence of individuals, taking into account the perceived difficulties in performing some activities (dressing, including putting on socks and shoes, walking across the room, bathing or showering, eating, such as cutting up food, getting in and out of bed, using the toilet, including getting up or down). This scale distinguishes between individuals who reported having one or more difficulties in basic activities of daily living, from those who stated no functional limitation.

The second variable, related to physical health, evaluates limitations in terms of mobility level, i.e. the ability to walk 100 metres; sit down for around two hours; get up from a chair after having been seated for some time; climbing several flights of stairs without resting; climbing a flight of stairs without resting; leaning, kneeling or crouching; placing or raising the arms above shoulder level; pulling or pushing large objects; lifting or carrying weights over 5 kilograms; picking up a small coin from the top of a table. This last variable distinguishes individuals who do not present any mobility limitations from those who have one or more limitations.

Macro-social-variables: country of residence, that distinguishes between individuals residing in Portugal and Estonia. In the regression analyses, Portugal was considered to be the reference category because it is the country where there are lower rates of Internet use.

## Results and discussion

Individuals resident in Portugal and Estonia, aged 50 years and older, who use the Internet are a minority group. In Portugal only 15.2% of respondents regularly use this technology. In Estonia the percentage of Internet users was 35.4%.

Table 1 shows the descriptive analyses of the socio-demographic, economic and health characteristics of Internet users and non-users in the whole sample.

**Table 1:** Characteristics of Internet users and non-users in the sample.

	Users	Non-users	p value	χ^2^*/t*	Kurtosis/Skewness	Cohen's *d /Phi*
**Average age** (SD)	60.07 (SD 8.337)	67.14 (SD 10.163)	p < .001	43.305	-.762/.304	1.002
**Gender**			p < .001	5.191		-.024
Female (%)	40.70%	59.30%				
Male (%)	59.30%	40.70%				
**Years of schooling—average** and standard deviation (in years)	11.76 (SD 4.720)	5.31 (SD 3. 355)	p < .001	-50.959	-.383/.021	-1.167
**Perception of financial situation**			p < .001	255.597		.172
Positive financial situation (%)	61.1%	41.70%				
Negative financial situation (%)	38.90%	58.30%				
**Mental health (Euro-D)**			p < .001	142.195		-.130
Exhibit depressive symptoms (%)	24.60%	39.60%				
Don’t exhibit depressive symptoms (%)	75.40%	60.40%				
**Functional limitations (ADL)**			p < .001	212.682		-.157
1+ ADL limitations (%)	8.30%	17.40%				
Without limitations (%)	91.7%	82.60%				
**Mobility limitations**			p < .001	437.028		-.225
1+ mobility limitations (%)	43%	55%				
No limitations (%)	57%	45%				

Source: SHARE wave 4, version 1.1.1 weighted data. N (non-weighted): Users = 2656; Non-users = 5983

Our results are consistent with findings from other studies, that suggest the existence of differences between the groups of Internet users and non-users according to age, gender, years of schooling, financial status, mental health (symptoms of depression) and physical health (functional capacity and mobility) [15,17,48].

The average ages of the two groups (Internet users and non-users) are statistically different (Internet users have 60.07 years on average, while non-users have 67.14 years) with large effect size, in line with different studies [16,25,27,38].

The group of Internet users is mainly composed of males (59.30%) while the group of non-users is essentially composed of females (with small effect size). With regard to education, on average, Internet users have a higher number of years of schooling (on average, 11.76 years for users, compared to 5.31 years for non-users, with large effect size) like found in other studies [17,19,25,26,49].

Internet users also report having fewer financial problems: 61.1% state that they can meet monthly expenses without difficulty, compared to 41.7% for non-users (with small-medium effect size).

The group of Internet users is positively differentiated from the group of non-users, with regard to mental and physical health, with fewer symptoms of depression, fewer functional limitations in performing daily activities and fewer limitations on the level of mobility (with small-medium effect size).

After carrying out this comparative descriptive analysis of Internet users and non-users, residing in the two countries, a logistic regression analysis was undertaken, to assess whether, after controlling for the effect of the various individual variables, differences still persist, related to the macro-social aspects of the two countries (Table 2).

**Table 2:** Determinants of Internet use, according to the characteristics of individuals aged 50+ years, living in Portugal and Estonia.

Variables	B	OR (95% CI)
**Sociodemographic and economic characteristics**		
Age	-.109	.897 (.890 -.904)***
Gender (Female)	-.058	.943 (.836–1.065)
Years of schooling	.314	1.369 (1.343–1.396)***
Perception of financial situation (positive perception)	.660	1.934 (1.711–2.185)***
**Health**		
Euro-D (≤ 3 symptoms of depression)	-.157	.855 (.750-.973)*
ADL (1+ limitation)	-.234	.791 (.647-.967)*
Mobility (1+mobility limitations)	-.161	.852 (.747-.971)*
**Country of Residence**		
Portugal	.575	1.777 (1.490–2.119)***
Constant	2.439	
Nagelkerke R Square = .462 p < .001		

*p < .05;

**p < .01;

***p < .001

Source: SHARE wave 4, non-weighted data. N (non-weighted) = 8283

The socio-demographic, economic, health and macro-social variables entered in the regression model explain 46% of the variance in Internet use by individuals aged 50 years or more.

We will first discuss the individual variables and then the macro-social variables, which constitute the main goal for this study. With regard to the individual variables, there is an inverse relationship between age and Internet use. The probability of using the Internet decreases by 10.3% for each year of additional age (OR = .897; 95% CI: .890 to .904). This is consistent with previous studies [16,25,27,38].

Some studies on gender have identified the persistence of inequalities in the use of ICT between male and female senior citizens [15,50]. On the other hand, in contrast to these studies, other studies suggest that the number of females using the Internet is tending to increase [51], moving towards growing gender parity [52–54]. Indeed, our results are consistent with the findings of these recent research studies, since we found no statistically significant gender differences, in relation to the probabilities of Internet use (p = .345).

The level of education, as expected, is strongly associated with Internet use, wherein individuals with more years of schooling tend to have a higher level of Internet use. These results mirror the results obtained in other studies, that have highlighted the importance of education in determining levels of Internet use amongst senior citizens [17,19,25,26,49]. Also, as was expected, and in accordance with the literature review [17,18,26], absence of economic difficulties, is associated with a higher probability of using this technology, which implies that cost of computer equipment and the price of Internet access continue to be significant factors of discrimination. Similarly, with regard to health, our results are consistent with previous research, that establishes a correlation between the existence of health problems with lower Internet use [17,25,27]. Hence, European citizens aged 50 years and more in the two countries under analysis that have significant symptoms of depression, i.e. 3 or more symptoms of depression in the EURO-D scale, are 18.5% less likely to use the Internet (OR = .855; 95% CI: .750 to .973) compared to those with under 3 symptoms. In turn, individuals who have limitations in performing daily activities are also distinguished from those who do not have these limitations, since the former are 20.9% less likely to use the Internet (OR = .791, 95% CI: .647 to .967). Also people who have one or more mobility limitations are 14.8% less likely to use this technology (OR = .852; 95% CI: from .7427 to .971) than those who don’t have such limitations.

Other studies have also shown a relation between physical or health problems and technology [27].

Finally, focusing on the main goal of this study, the country of residence constitutes an important factor for Internet use. Although Portugal has fewer Internet users than Estonia, once the effects of individual variables have been controlled for, the residence of persons aged 50 years and over in Portugal is actually associated with a higher probability of Internet use.

In Portugal, the majority of individuals aged 50 years and over tend to have a set of socio-demographic characteristics that are strongly related to the profile of an Internet non-user, because, in addition to health-related limitations which are an issue to consider [55], older Portuguese citizens, especially those who spent their childhood and youth under the dictatorship regime, had limited or no educational opportunities in the past, and today have limited pensions [56] because they only made limited pension contributions, which thus hinders their access to paid Internet services. However, the probabilities of Portuguese citizens aged 50 years and over using the Internet, are actually higher than in Estonia, after having controlled for the socio-demographic and health characteristics of the population in the study. These results are particularly interesting and relevant for definition of public policies and may have been affected by the significant investments made in Portugal over the past decade in technology programs [57]; adult education (e.g. the New Opportunities programme, EFA courses, etc.); the provision of specific IT training by senior citizen universities, parish councils and NPOs, often available free of charge, and the creation of public spaces for free Internet access. Another factor that has probably contributed to this result is the incentive to use the Internet, which has been achieved in Portugal, in particular, through increasing computerisation of administrative acts (eGovernment).

The results presented herein highlight the need to analyse e-inclusion, taking into consideration not only socio-demographic, economic and health variables, at an individual level, but also the macro-social variables related to the country of residence. In this sense, future research should include, in addition to the variables that are traditionally included in studies on technology-use, variables related to the importance that different public policies may have on reducing the "digital divide" and fostering the e-inclusion of individuals aged 50 years and over.

This study presents certain limitations that must be taken into account in future research. SHARE’s database, which is of great value for carrying out comparative analyses of Europeans aged 50 years and over, has significant information gaps in relation to Internet use by these individuals. It would therefore be advisable to collect data on other variables associated to the "digital divide", such as different types of Internet use and the frequency with which individuals use this technology in the various countries participating in the project. It would also be advisable for future research with SHARE data to include variables of the user acceptance of information technology models well integrated in the Unified Theory of Acceptance and Use of Technology (UTAUT) [58] and its extension UTAUT2 [59]. This information would make it possible to go further the results of this study, which points to the importance of the macro-social context and public policies, in the construction of a more inclusive Information Society.

## Supporting information

S1 Table Quantitative studies analysing the influence of socio-demographic characteristics on technology use. (DOCX)
