# Supplementary material for: E-inclusion: Beyond individual socio-demographic characteristics
Source: PLoS One. 2017 Sep 14;12(9):e0184545. doi: 10.1371/journal.pone.0184545 (PMC5598973; doi:10.1371/journal.pone.0184545)
Supplement: S1 Table — (DOCX) [file pone.0184545.s001.docx]

**Supporting Information**

**S1 Table. Quantitative studies analysing the influence of sociodemographic characteristics on technology use**

| **Author** | **Method** | **Sample (N)** | **Location** | **Analysis** | **Variables** |
| --- | --- | --- | --- | --- | --- |
| Yu, Ellison, McCammon, & Langa (2016) | Survey | 18851 | USA | Logistic regression and interactions | Internet; SNS; age; gender; race/ ethnicity.; years of education; marital status; employment status; income and wealth; cognitive functioning, self-rated health and diversity of Internet activities |
| Friemel (2014) | Survey | 1105 | Switzerland | Logistic regression | Internet;age; gender; languag; education; income; general technical interest; social network; pre-retirement computer use; reasons for not using the Internet |
| Stoica (2015) | Survey | 728 | Iasi | Descriptive analyzes | Age; owning an IT device; internet access,; reasons for not having an Internet access at home; place of Internet access; frequency of Internet use; computer skills; internet skills and reasons for accessing Internet |
| Lissitsa & Chachashvili-Bolotin (2015) | Survey | 73523 (2068 respondents aged 65+). | Israel | Logistic regression and Ordinal regression | Ethnicity; age; gender; religiosity; marital status; number of children; area of residence education; income; employment, hebrew language proficiency; using the internet; human capital; social capital, physical or health problems |
| Van Deursen & van Dijk (2014) | Survey | 2010= 1418  2011= 1114 2012= 1224  2013= 1125 | Netherlands | Multiple linear regression and interactions | Age; education gender; internet usage; operational skills; formal skills; information skills; strategic skills |
| Casado-Muñoz, Lezcano & Rodríguez-Conde (2015) | Survey | 419 | Spanish | Descriptive analyzes | Age; gender; computer at home; computer ownership; use of the computer, internet acess; place of access; internet activities. motives and needs of the use of computers and Internet |
| Aroldi, Colombo & Carlo (2015) | Survey | 900 | Italy | Descriptive analyzes and Clusters | Family relations; health status; lei sure time and cultural consumption; any past or present working condition; participation in any kind of volunteering or socio-political activities; social capital and social solidarity; family networks and friendships; values; representation of the elderly condition, economic status and media use |
| Gilleard, Jones & Higgs (2015) | Survey | 2000=18631  2006 =2198  2009 = 18102 | UK | Logistic regression | Gender; housing tenure; health; income; household ownership of a PC; educational status; presence versus absence of under 16s in the household and household size; access to a mobile phone |
| Correa, Straubhaar, Chen, & Spence (2013) | Survey | 1701 | Austin (USA) | Logistic and hierarchical regression | Age; gender; education; income; internet learning children as internet brokers; internet self-efficacy; online activities |
| Agudo-Prado, Pascual-Sevillano, & Fombona-Cadavieco (2012) | Survey and discussion groups | 215 | Spain | Descriptive analyzes | Gender; technological resources; use that the elderly make of ICT; education; information, communication and entertainment. |
| Wei (2012) | Survey | 2251 | USA | Regression | Gender; age; race; levels of education and income; Internet activities; political communication and political participation |
| Van Deursen, van Dijk, & Peters (2011) | Performance tests and survey | Study 1 =109 Study 2= 109 Study 3 = 88 | Netherlands | Structural equation modeling | Gender; age; educational level of attainment; internet experience; hours online; operational skills; formal skills ; information skills; strategic skills |
| Lee, Chul-Joo (2009) | Survey | 2489 | USA | Multiple regression analyses | Age; gender; formal education; income; race, general health knowledge; internet use for health information; internet engagement |
| Loges & Jung (2001) | Survey | 1809 | USA | Regression | Age; gender; income; education; employment; Internet connectedness index; history and contexto; scope and intensity; centrality |
| Diño & de Guzman (2015) | Survey | 82 | Northern Philippines | Structural Equation Modeling | Gender; UTAUT |
| Ramón-Jerónimo, Peral-Peral, & Arenas-Gaitán (2013) | Survey | 492 | Spain | Multigroup approach of structural equations models | Age; education; gender retirement;TAM |
| Selwyn, Gorard, Furlong, & MaddenM (2003) | Survey | 352 | England and Wales | Descriptive analyzes | Gender; age; marital-status; health-status; education; access to computers; sources of ICT support; use of computers and the Internet and reason for not using ICT |
| Wong, Chen, Lee & Fung, Law (2013) | Survey | 245 | Hong Kong. | Descriptive analyzes | Gender; age; education; knowledge of computers; empowerment |
| Jung et al., (2010) | Survey | 91 | Los Angeles | Logistic regression | Age; gender; education; income; aging anxiety índex; computer experience; belonging to the neighborhood; belonging to the senior center; computer self-efficacy índex; multidimensional scale of perceived social support index |
